# Supplementary figures and images for: Disruption of macrophage cell volume drives inflammatory responses and type I interferon signaling
Source: J Cell Biol. 2026 May 7;225(6):e202411133. doi: 10.1083/jcb.202411133 (PMC13151915; doi:10.1083/jcb.202411133)

Source data Fig 2I

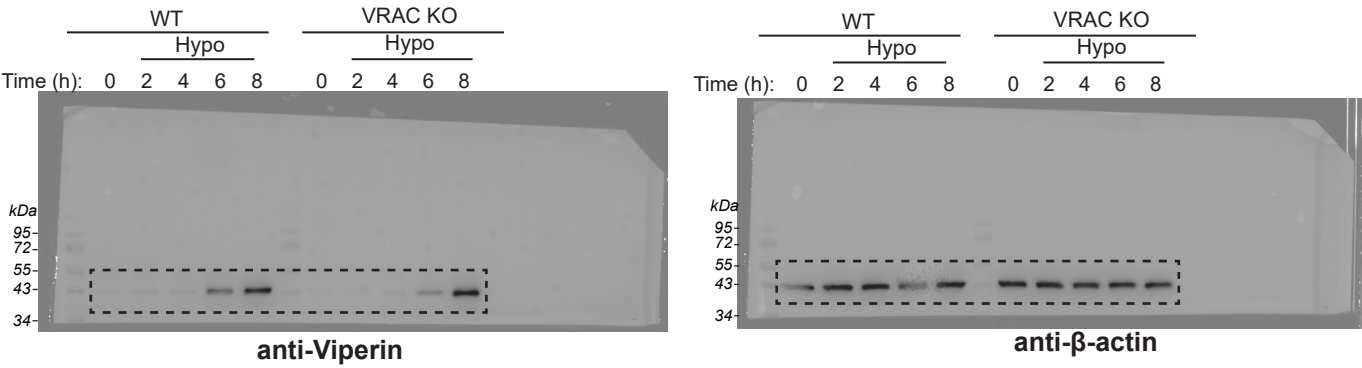

Source data Fig 2M

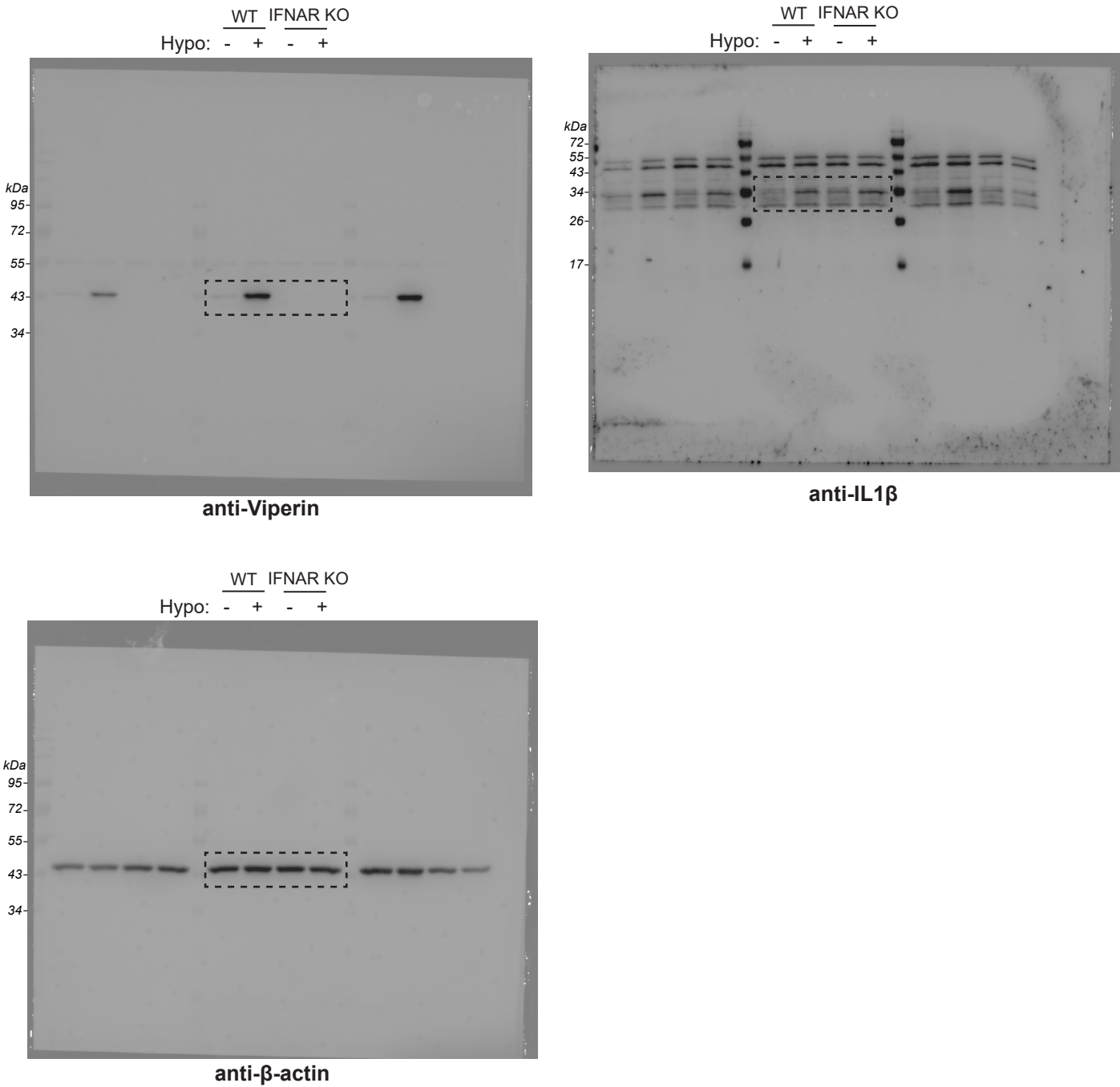

Supplement: SourceData F2 — is the source file for Fig. 2. [file jcb_202411133_sourcedataf2.pdf]

Source data Fig 3A

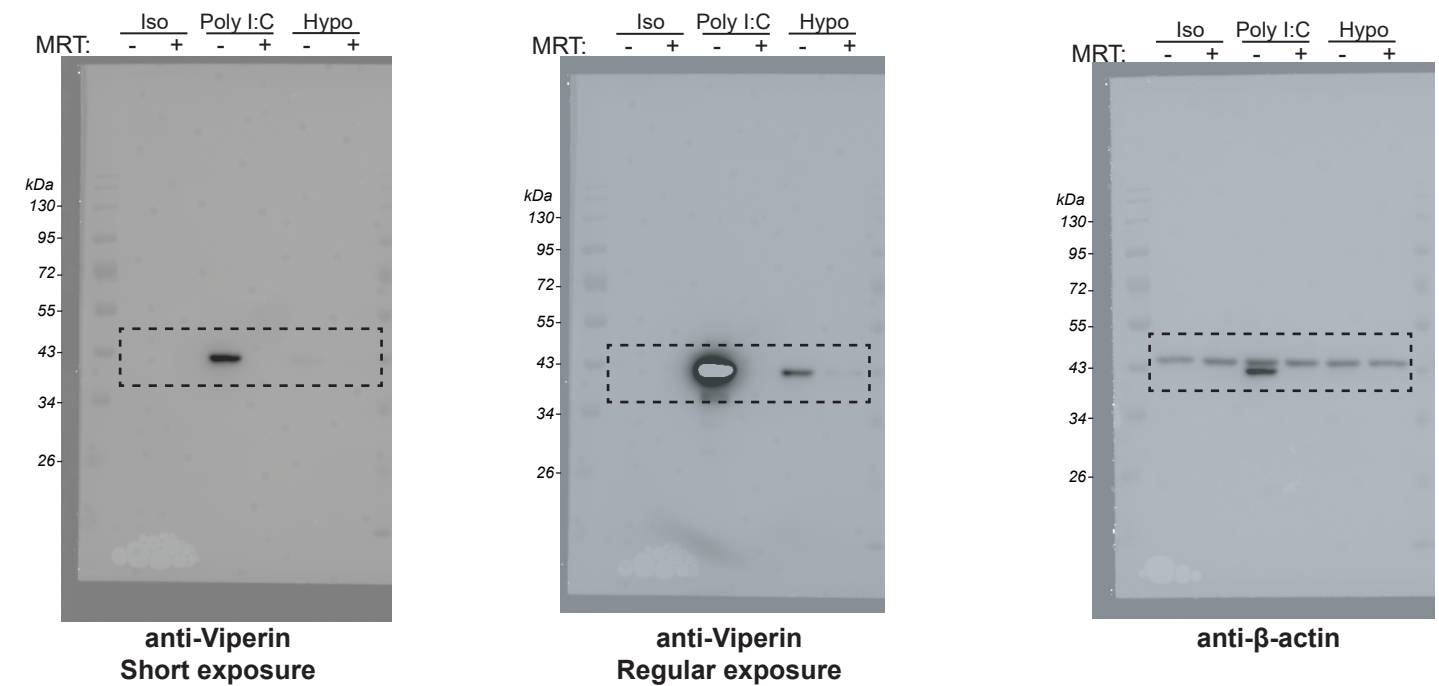

Source data Fig 3C

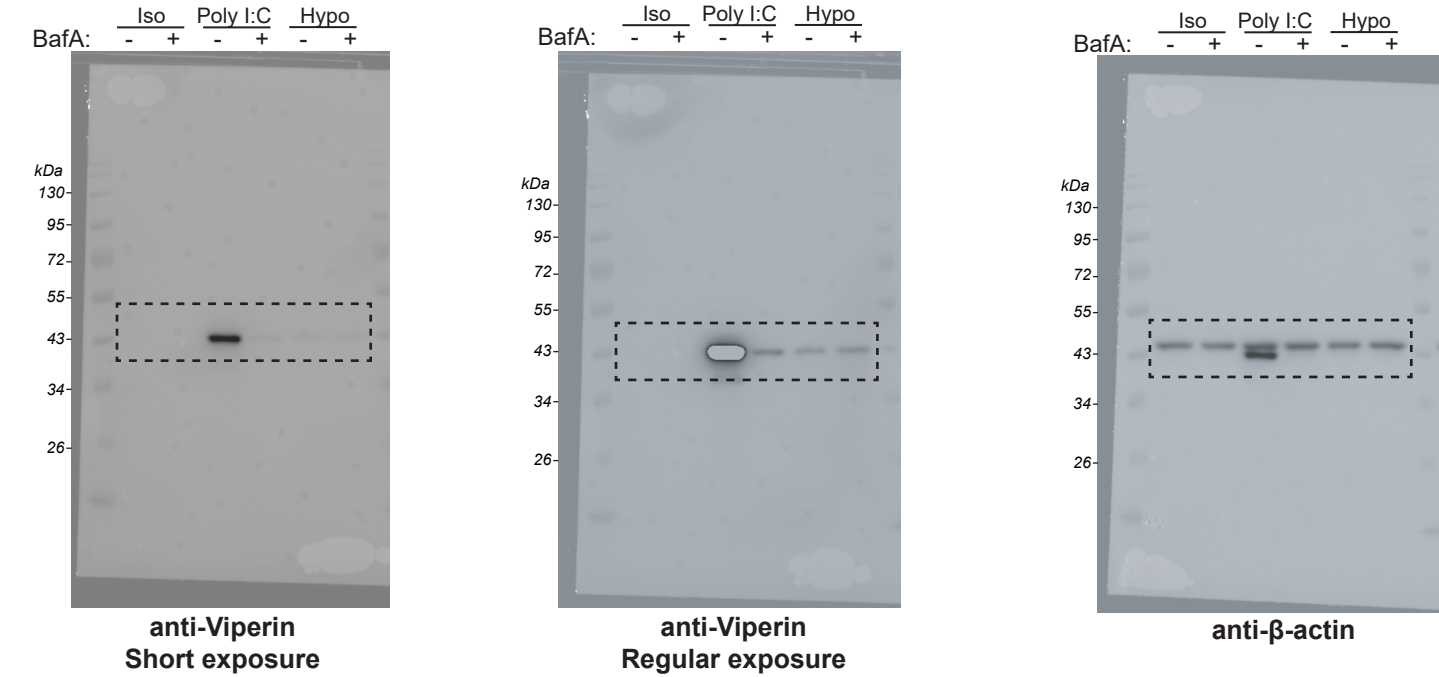

Supplement: SourceData F3 — is the source file for Fig. 3. [file jcb_202411133_sourcedataf3.pdf]

Source data Fig 4A

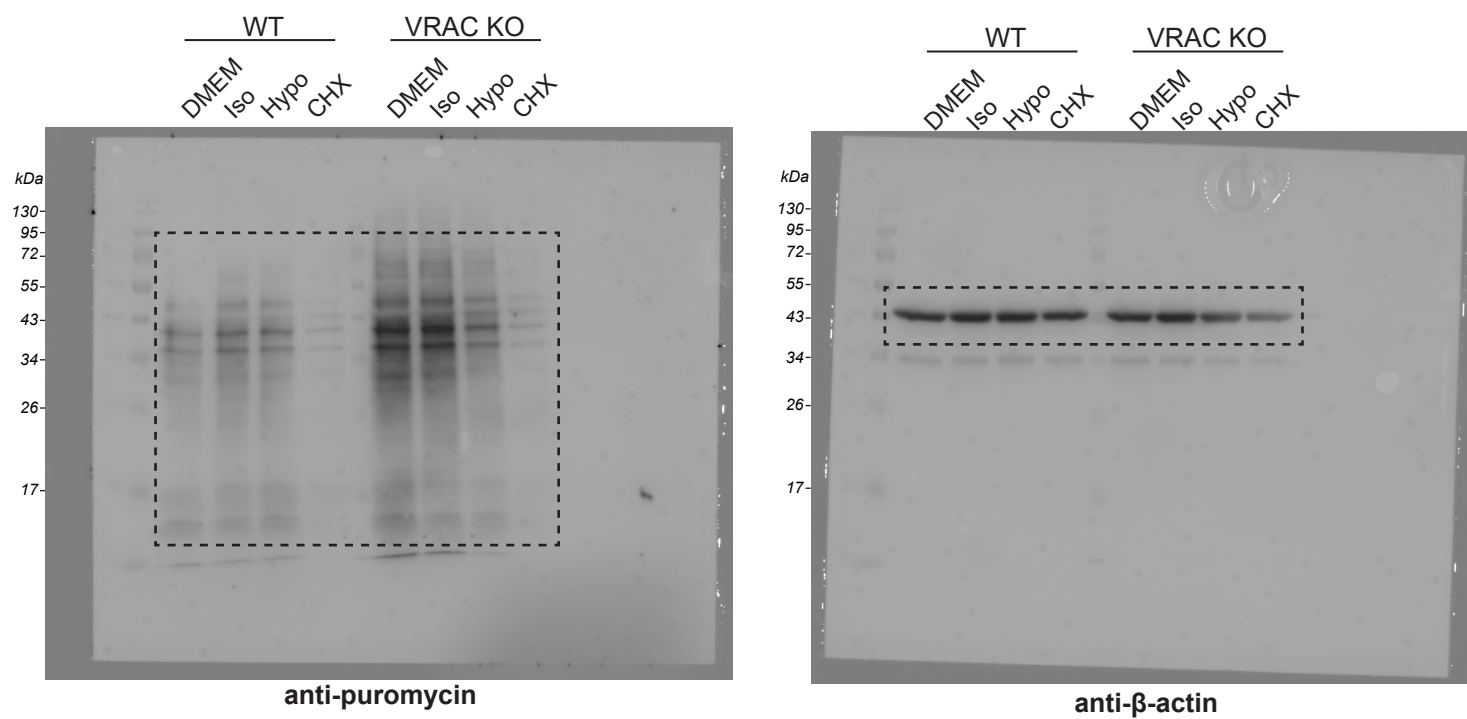

Supplement: SourceData F4 — is the source file for Fig. 4. [file jcb_202411133_sourcedataf4.pdf]

Source data Fig 5E

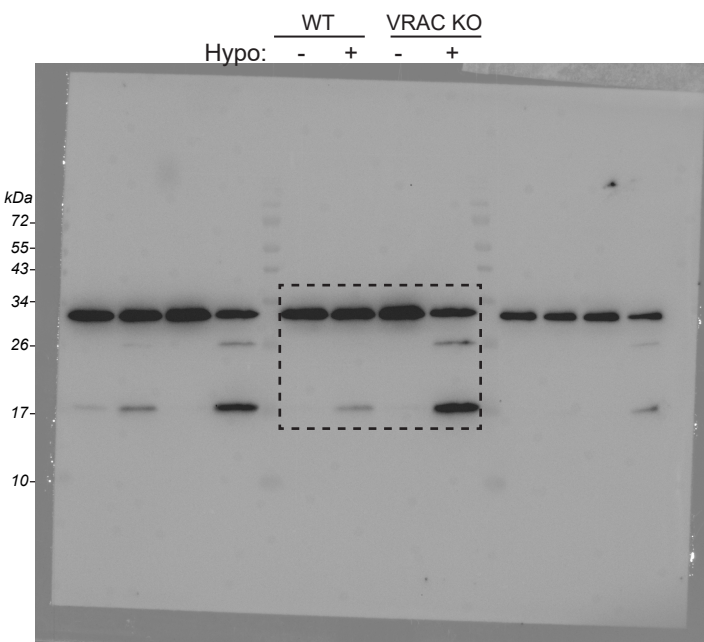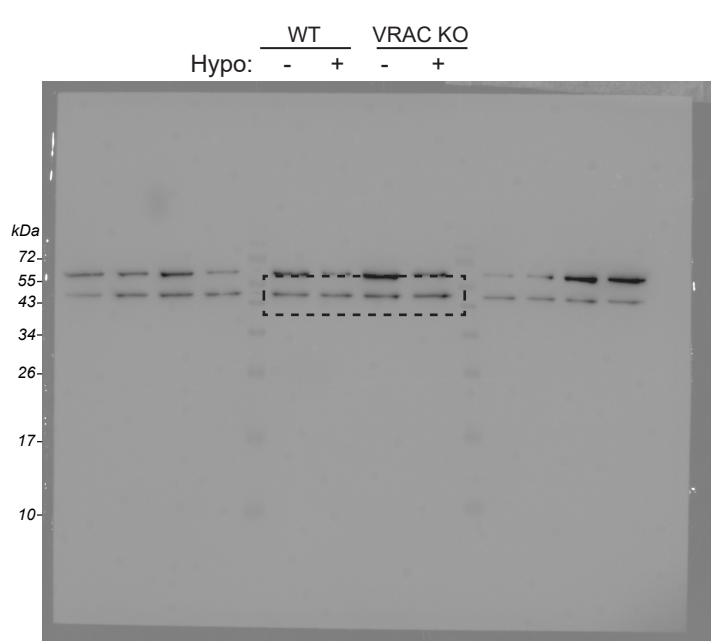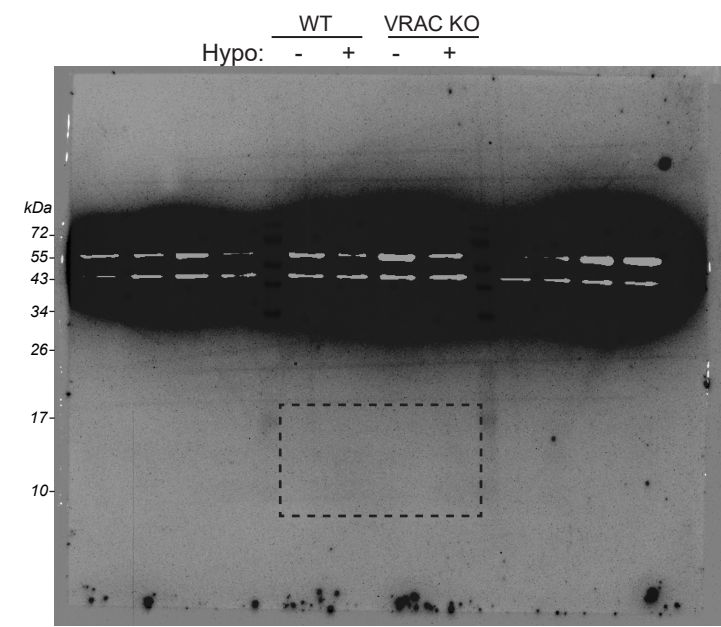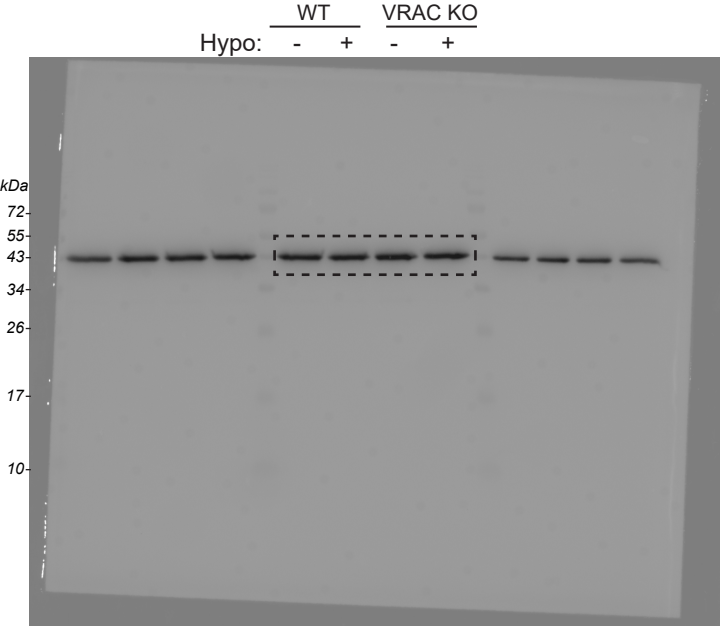

Source data Fig 5l

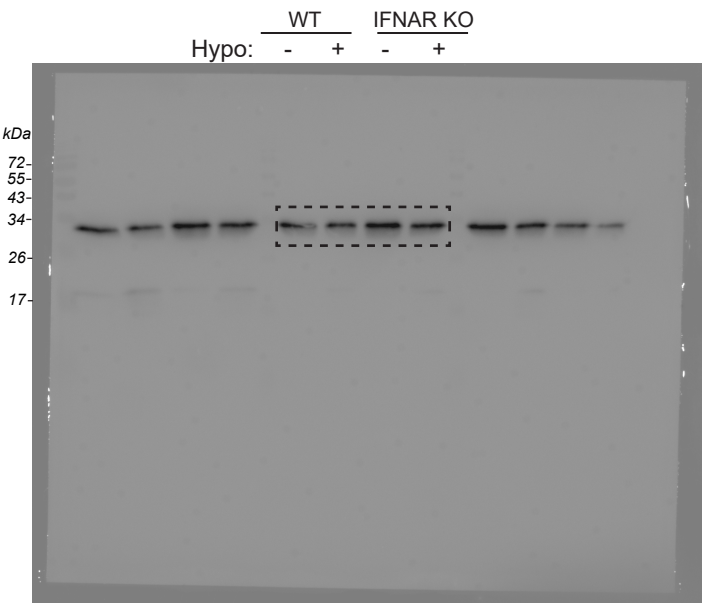

anti-Caspase-3

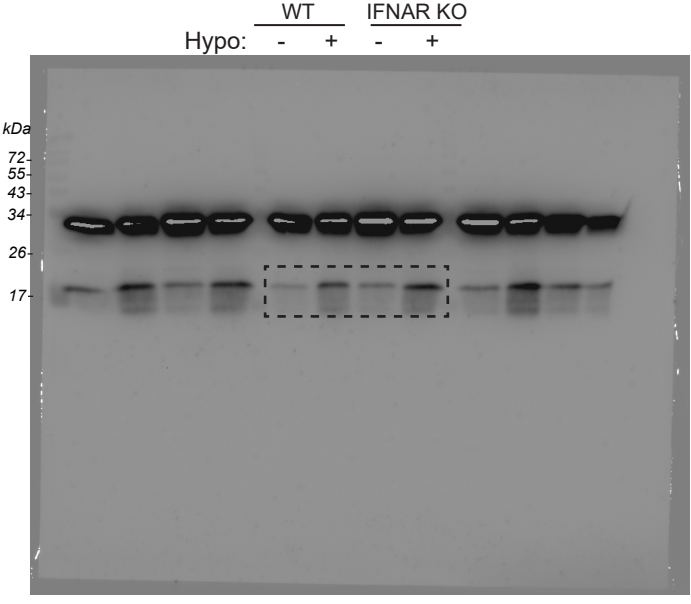

anti-Caspase-3  
Prolonged exposure

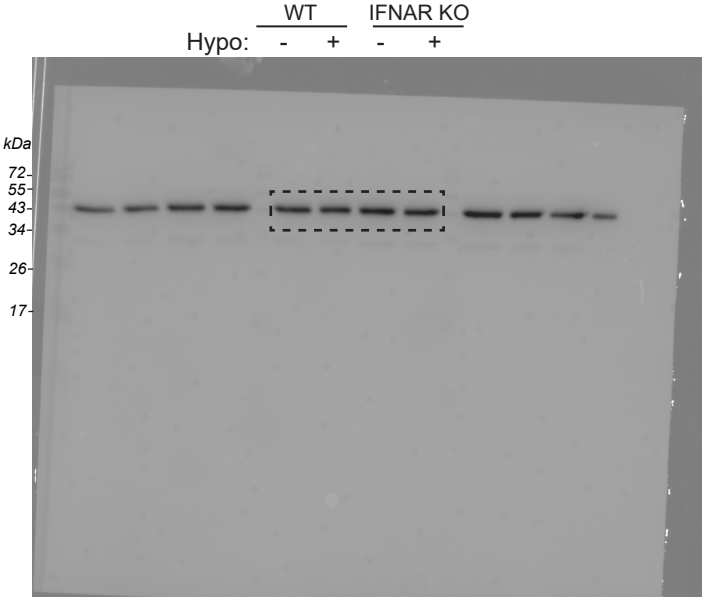

anti-β-actin

Supplement: SourceData F5 — is the source file for Fig. 5. [file jcb_202411133_sourcedataf5.pdf]

Source data Fig 6F

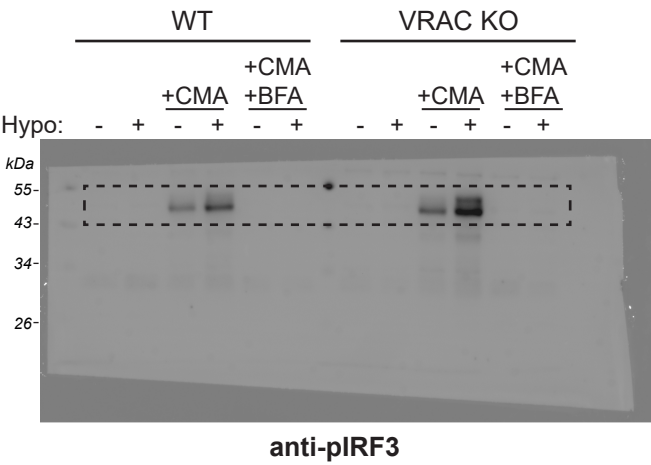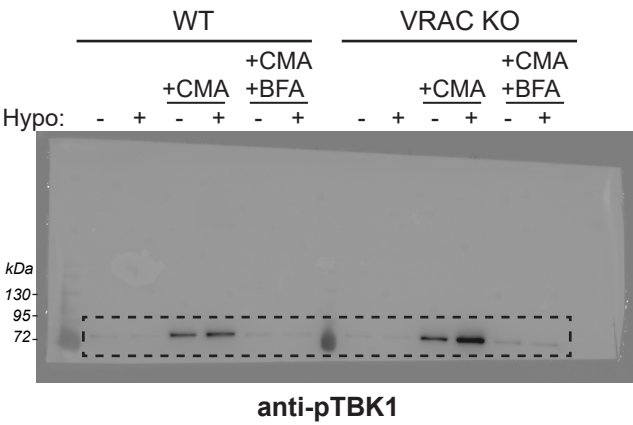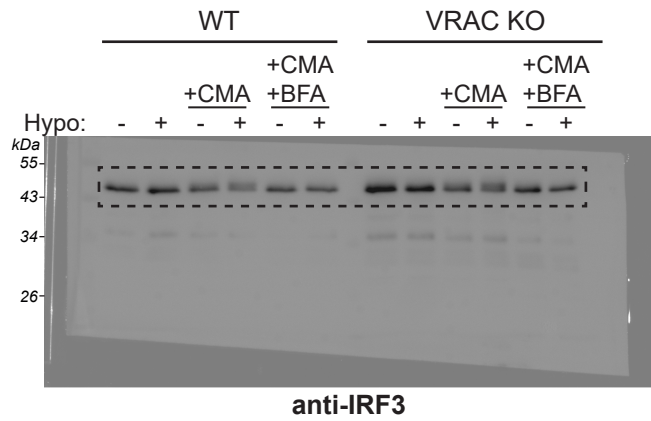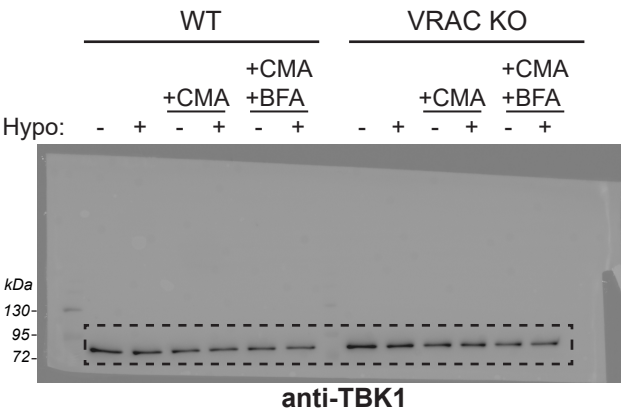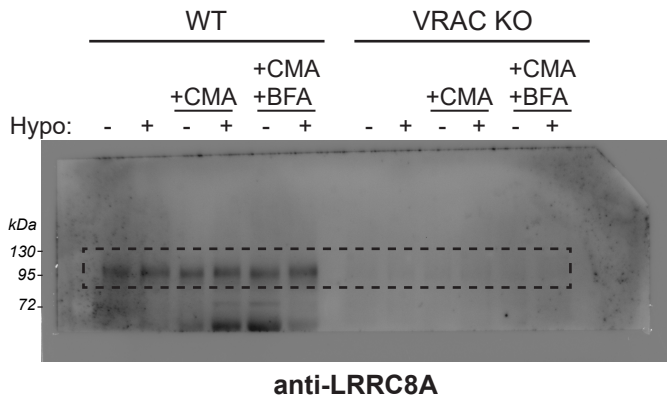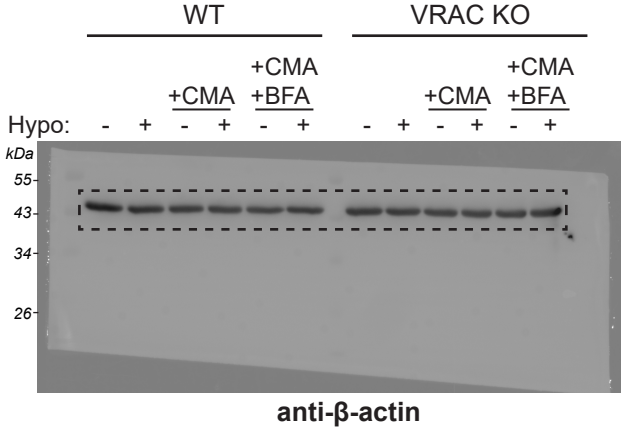

Source data Fig 6I

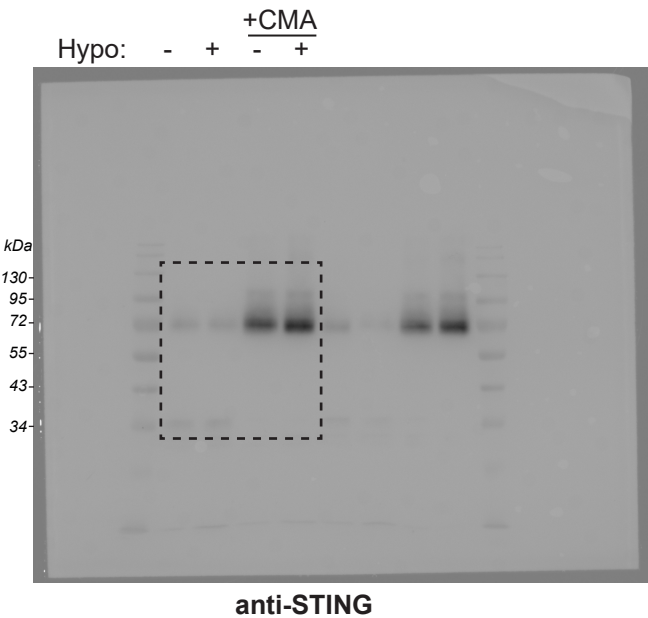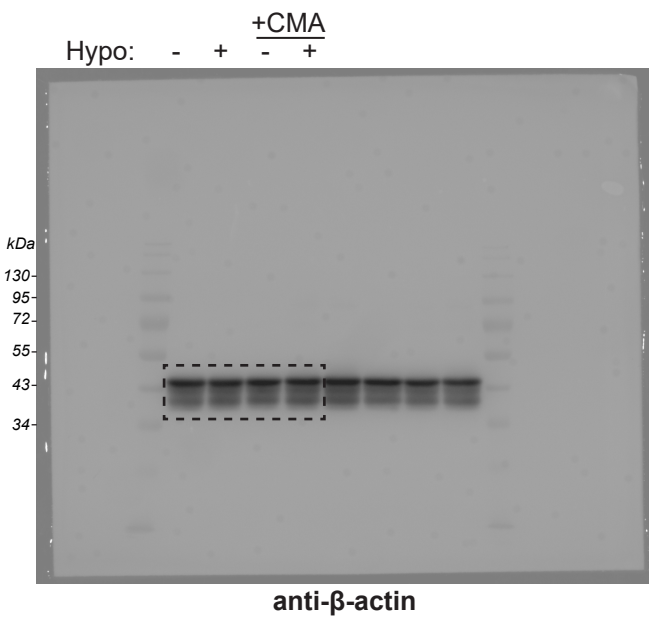

Source data Fig 6N

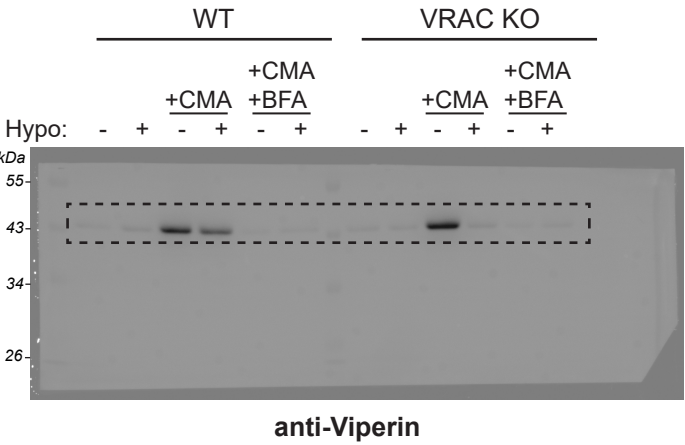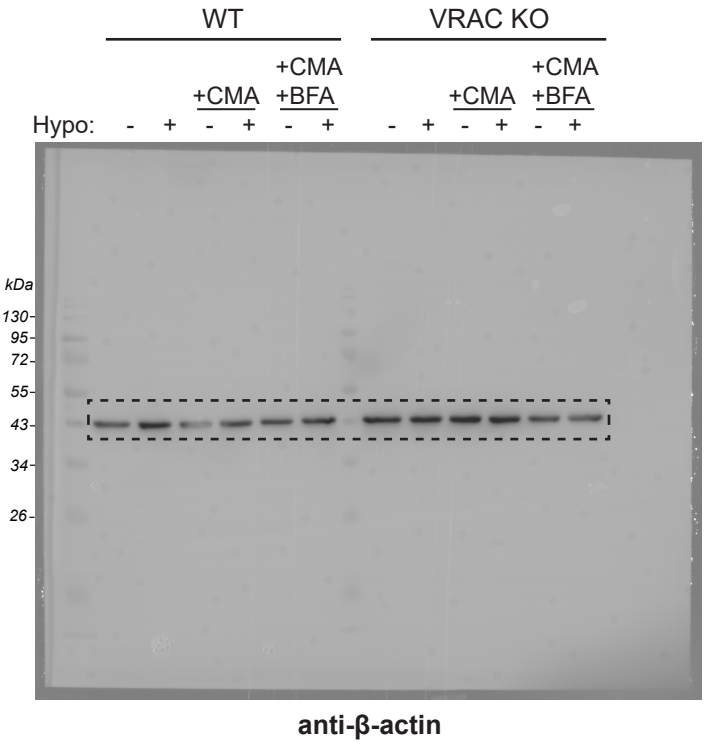

Supplement: SourceData F6 — is the source file for Fig. 6. [file jcb_202411133_sourcedataf6.pdf]

Source data Fig 8H

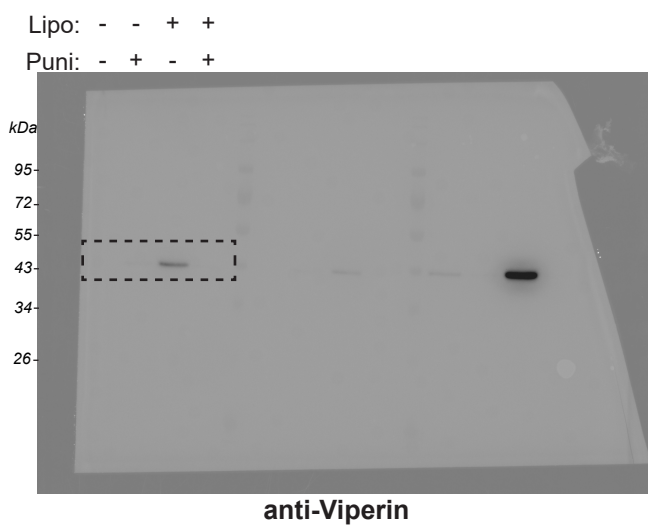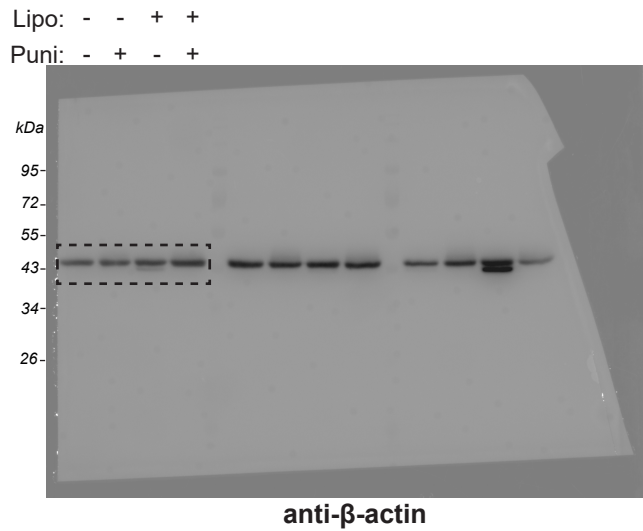

Supplement: SourceData F8 — is the source file for Fig. 8. [file jcb_202411133_sourcedataf8.pdf]
