# Supplementary material for: Disruption of macrophage cell volume drives inflammatory responses and type I interferon signaling
Source: J Cell Biol. 2026 May 7;225(6):e202411133. doi: 10.1083/jcb.202411133 (PMC13151915; doi:10.1083/jcb.202411133)
Supplement: SourceData FS2 — is the source file for Fig. S2. [file jcb_202411133_sourcedatafs2.pdf]

Source data Supplementary Fig 2D

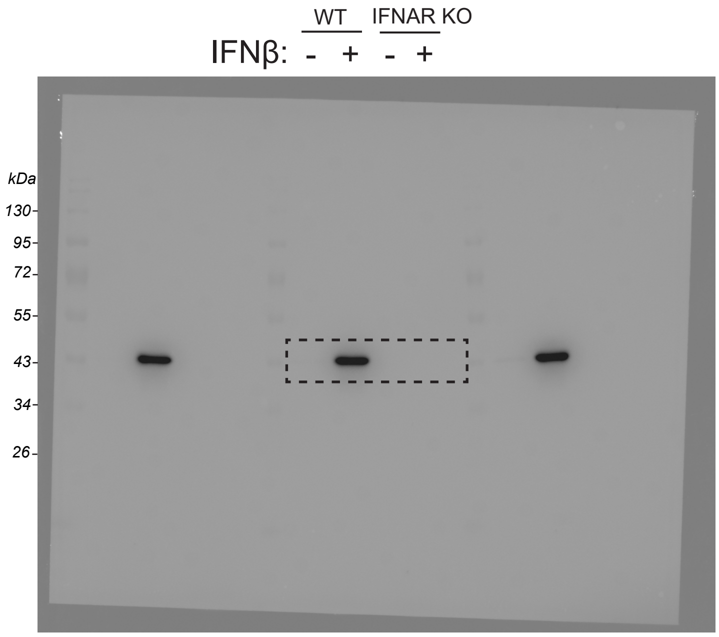

anti-Viperin

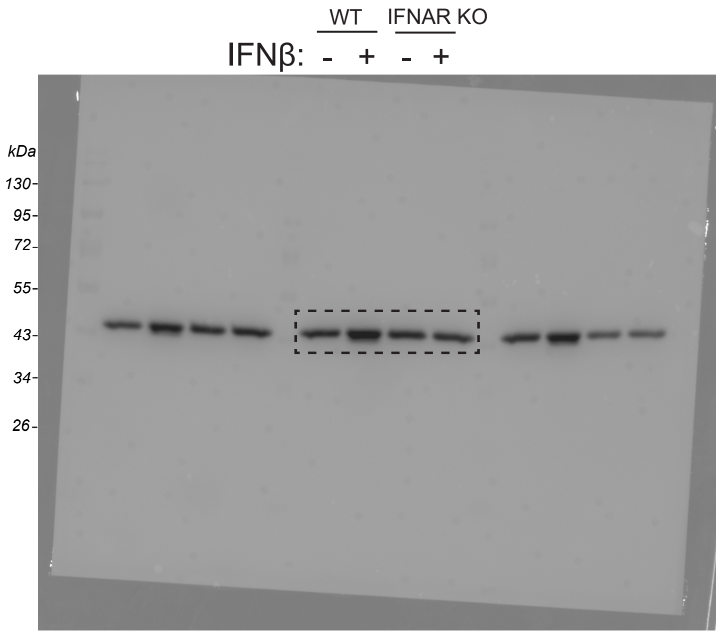

anti-β-actin

Source data Supplementary Fig 2E

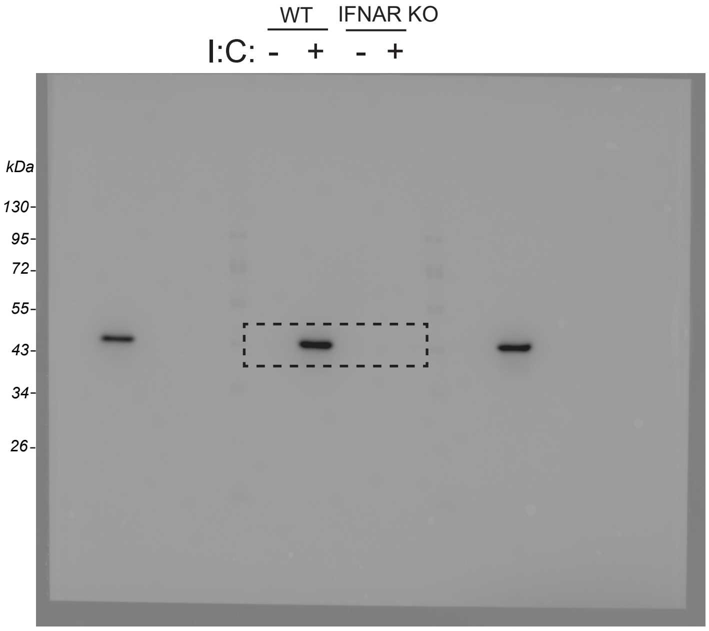

anti-Viperin

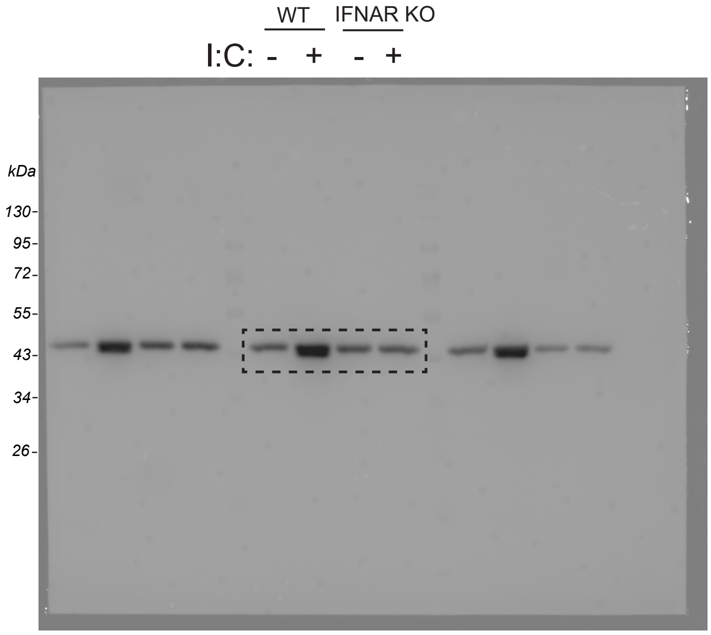

anti-β-actin
